# [Retracted] Protection of rat intestinal epithelial cells from ischemia/reperfusion injury by (D‑Ala2, D‑Leu5)‑enkephalin through inhibition of the MKK7‑JNK signaling pathway

**DOI:** 10.3892/mmr.2024.13403

**Published:** 2024-11-25

**Authors:** Zhenran Wang, Bo Tang, Fang Tang, Yang Li, Guangyu Zhang, Li Zhong, Chencheng Dong, Songqing He

Mol Med Rep 12: 4079–4088, 2015; DOI: 10.3892/mmr.2015.3991

Following the publication of this paper, it was drawn to the Editors’ attention by a concerned reader that certain of the cell apoptotic assay data shown in Fig. 2A on p. 4082 were strikingly similar to data appearing in different form in another article written by different authors at different research institutes that had already been published in the journal *Life Sciences* prior to the submission of this paper to *Molecular Medicine Reports*. In view of the fact that the abovementioned data had already apparently been published previously, the Editor of *Molecular Medicine Reports* has decided that this paper should be retracted from the Journal. The authors were asked for an explanation to account for these concerns, but the Editorial Office did not receive a reply. The Editor apologizes to the readership for any inconvenience caused.

